# Characterization of the Sexually Dimorphic *fruitless* Neurons That Regulate Copulation Duration

**DOI:** 10.3389/fphys.2018.00780

**Published:** 2018-06-25

**Authors:** Shreyas Jois, Yick Bun Chan, Maria Paz Fernandez, Adelaine Kwun-Wai Leung

**Affiliations:** ^1^Department of Veterinary Biomedical Sciences, Western College of Veterinary Medicine, University of Saskatchewan, Saskatoon, SK, Canada; ^2^Department of Neurobiology, Harvard Medical School, Boston, MA, United States

**Keywords:** courtship, copulation duration, *Drosophila melanogaster*, fruitless, serotonergic, dopaminergic

## Abstract

Male courtship in *Drosophila melanogaster* is a sexually dimorphic innate behavior that is hardwired in the nervous system. Understanding the neural mechanism of courtship behavior requires the anatomical and functional characterization of all the neurons involved. Courtship involves a series of distinctive behavioral patterns, culminating in the final copulation step, where sperms from the male are transferred to the female. The duration of this process is tightly controlled by multiple genes. The *fruitless* (*fru*) gene is one of the factors that regulate the duration of copulation. Using several intersectional genetic combinations to restrict the labeling of GAL4 lines, we found that a subset of a serotonergic cluster of *fru* neurons co-express the dopamine-synthesizing enzyme, tyrosine hydroxylase, and provide behavioral and immunological evidence that these neurons are involved in the regulation of copulation duration.

## Introduction

Male courtship in *Drosophila melanogaster* is an innate behavior that involves several distinctive behavioral patterns. As the neural circuit responsible for courtship is hardwired genetically in the nervous system, courtship behavior is an excellent model for investigating the connection between genes, neural circuits, and behavior. Male courtship is initiated upon the recognition of a female. The male fly performs a courtship “ritual” that consists of a sequence of steps, including orientation, chasing, tapping, singing, and licking (Pavlou and Goodwin, 2013; Yamamoto and Koganezawa, 2013; Yamamoto et al., 2014). Once the female is receptive to the male, she slows down, and opens her vaginal plate (Rezaval et al., 2012, 2014). The male subsequently bends his abdomen, and attempts to copulate (Pavlou and Goodwin, 2013; Yamamoto and Koganezawa, 2013; Yamamoto et al., 2014). The final copulation step requires an intricate neuronal coordination of the copulatory muscles, an integration of sensory signals from the genitalia (Pavlou et al., 2016), and signals for ejaculation at the reproductive organs (Tayler et al., 2012). The proper coordination of these neuronal components leads to a regulated copulation duration that ensures successful mating (Pavlou and Goodwin, 2013; Yamamoto and Koganezawa, 2013; Yamamoto et al., 2014).

The duration of copulation is species-specific and multifactorial. Many genetic mutants have been reported to have abnormal copulation duration (Yamamoto et al., 1997). Indeed, the *fru* gene has been identified as an important regulator of copulation duration. Compared to normal males, classic semi-fertile *fru* mutants demonstrate marked variability in the duration of copulation, with copulation being longer on average (Lee et al., 2001). This suggests that a population of *fru* neurons, with heterogeneous effects, controls copulation duration. *fru* is well known for its role in the development of the neural circuit that controls male courtship behavior (Demir and Dickson, 2005). The transcript from one of the four promoters of *fru* is alternatively spliced in a sex-specific manner to produce a functional Fru^M^ protein in males (Ryner et al., 1996). The expression of Fru^M^ in approximately 2000 neurons of the fly nervous system is essential for the proper development of the male courtship neural circuit (Lee et al., 2000). While most *fru*-positive neurons are present in both males and females, Fru^M^ is only expressed in males (Ryner et al., 1996). Sexual dimorphisms of *fru* neurons include cell count (Kimura et al., 2005; Cachero et al., 2010; Yu et al., 2010), the volume of synaptic structures (Stockinger et al., 2005; Cachero et al., 2010; Yu et al., 2010), and dendritic arborization patterns (Kimura et al., 2005, 2008; Datta et al., 2008; Cachero et al., 2010; Yu et al., 2010). Anatomical descriptions of *fru* neurons have largely focused on the brain, while those from the ventral nerve cord and the periphery are missing or incomplete (Cachero et al., 2010; Yu et al., 2010). Functionally, evidence is emerging on the roles of subsets of *fru* neurons in courtship initiation (Kimura et al., 2008; Kohatsu et al., 2011), song production (Rideout et al., 2007; von Philipsborn et al., 2011), sensory integration (Datta et al., 2008; Koganezawa et al., 2010), copulation initiation (Latham et al., 2013; Pavlou et al., 2016), sperm transfer (Tayler et al., 2012), and copulation duration (Tayler et al., 2012; Latham et al., 2013).

Intersectional genetic techniques provide the opportunity to examine specific subsets of neurons, and *Drosophila melanogaster* has a wide range of genetic tools available (Luan et al., 2006; Bohm et al., 2010; Ting et al., 2011). In this study, we generated a library of about 200 enhancer-trap lines with specific expression of the FLP recombinase (FLP) in neuronal tissues. This *FLP* library was combined with *fru-GAL4* to genetically dissect the circuit into smaller components, as only cells that express both GAL4 and FLP will be targeted. Using this strategy, we successfully restricted gene expression to a cluster of previously characterized sexually dimorphic serotonergic *fru* neurons in the abdominal ganglion. By combining the *FLP* library with other *GAL4* lines that specifically target different neurotransmitter systems, we provide immunochemical and behavioral evidence that lead to novel insights into the neurochemistry and behavioral function of these serotonergic *fru* neurons.

## Results

### Genetic Dissection of the Fruitless Circuit

**FIGURE 1:**
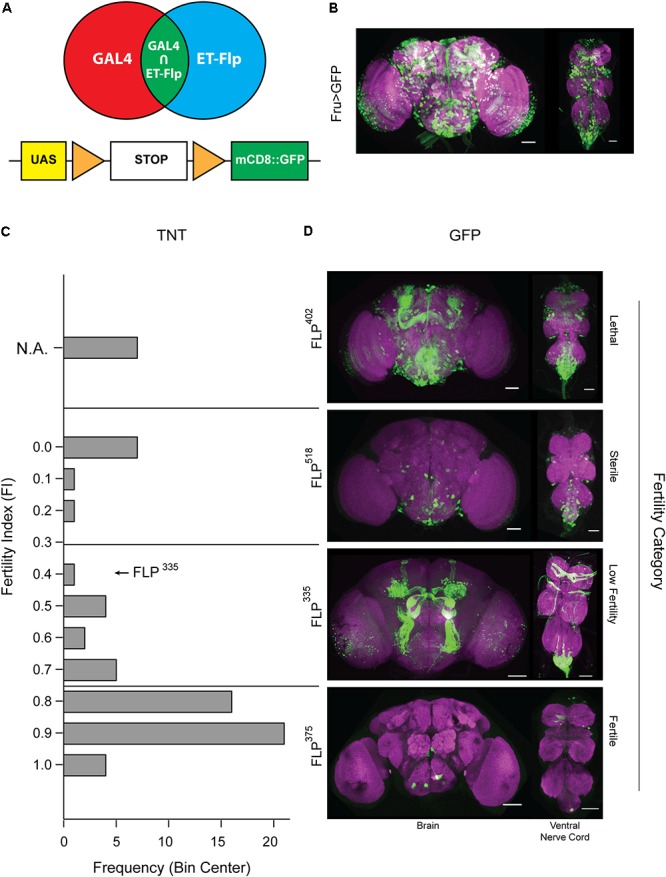
Restrictive labeling of the fru-Gal4 line using the *FLP* library. **(A)** Schematic representation of our intersectional genetics approach using *FLP* and *GAL4* to target *fru* subpopulations. **(B)** Expression pattern of the complete *fru* circuit in the CNS. **(C,D)** Tetanus toxin is expressed in *fru* neurons targeted by combining lines from the *FLP* library with *UAS>stop>TNT* and *fru-GAL4*. (Left Panel): Histogram depicting the fertility index distribution of the complete tetanus toxin screens containing 67 *FLP* lines that showed restricted GFP expression when combined with *UAS>stop>mCD8::GFP* and *fru-GAL4*. (Right Panel): Sample expression profile in each fertility category. Although the restricted *fru* expression profiles should be present in the full circuit image in **(B)**, it may not be apparent due to variation in intensity and overlapping signals. Tissues were stained with anti-mCD8 (green) and anti-nC82 (magenta). Scale Bar = 50 μm.

Over 2000 *fru* neurons are present in the adult male nervous system. These neurons have been shown to modulate nearly all aspects of the male courtship ritual. In order to identify restricted populations of *fru* neurons that are involved in the regulation of male copulatory behavior specifically, we used an intersectional genetic approach to combine the *FLP* lines that express FLP recombinase in neural tissues with a *GAL4* line that labels the *fru* circuit (*fru*-*GAL4*) (Stockinger et al., 2005) (**Figures 1A,B**). A total of 67 *FLP* lines showed restricted GFP expression in subsets of *fru* neurons (**Figure 1D**). As *fru* neurons are well established in the regulation of male courtship, we asked how disrupting specific subsets of this circuit would affect courtship behavior. By combining individual *FLP* lines with *fru-GAL4* and *UAS>stop>TNT*, we expressed TNT in specific subsets of *fru* neurons, thereby inactivating neural transmission from these neurons (Sweeney et al., 1995). All crosses produced progeny viable till eclosion. However, 7 *FLP* lines, that showed widespread FLP expression, produced males that died within 4 days after eclosion, suggesting that silencing the majority of *fru* neurons is lethal for adult males. Indeed, transgenic flies expressing TNT in all *fru* neurons (*UAS-TNT*; *fru-GAL4*) barely survive after eclosion (data not shown).

Next, we performed a fertility screen, and tested whether males with different subsets of the *fru* circuit silenced display courtship defects when paired with wild-type females. We began by quantifying the percentage of pairings that successfully produced progeny, or the FI. Based on the FI values, the *FLP* library was divided into three distinct fertility categories (sterile, low fertility, and normal fertility). The *FLP* lines belonging to the normal fertility category showed GFP expression in a small number of neurons scattered throughout the central nervous system (CNS). Silencing these neurons is not expected to have a pronounced effect that could be observed in the fertility screen. On the other hand, males generated from *FLP* lines belonging to the sterile category showed clear locomotion defects, suggesting that their sterility is not attributable to deficiencies specific to courtship. All sterile lines showed widespread expression of GFP, indicating that these *FLP* lines targeted rather large portions of the *fru* circuit. Finally, we hypothesized that the low fertility category is likely to contain *FLP* lines that target subsets of *fru* neurons pertaining to courtship (**Figures 1C,D**). Within this group, we selected several lines that showed consistent restricted expression of GFP in *fru* neurons. With these lines, we then performed more detailed courtship assays to determine what aspect of the courtship ritual is affected when these subsets of *fru* neurons are silenced.

### Identification of Subsets of Sexually Dimorphic *fru* Neurons That Impair Courtship and Copulatory Behaviors

**FIGURE 2:**
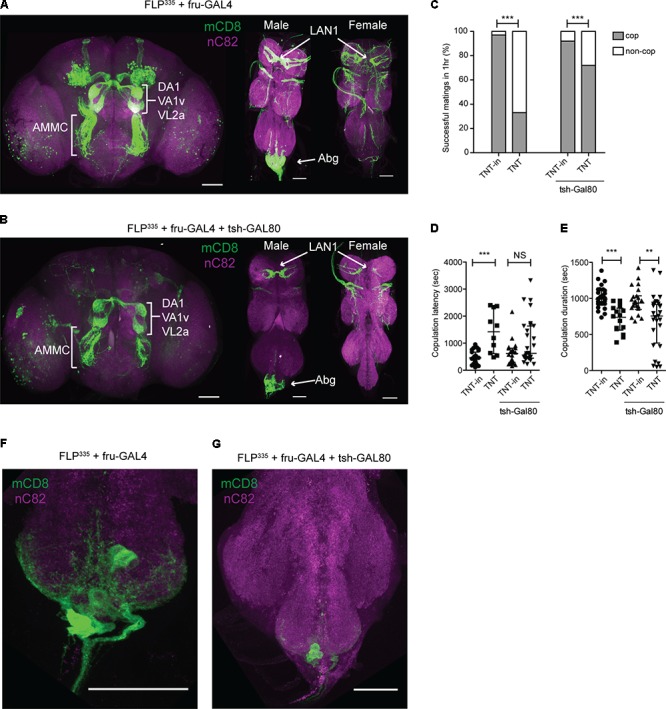
FLP^335^ restricted labeling of fru-Gal4. **(A)** Expression profile of FLP^335^ fru-GAL4 UAS>stop>mCD8::GFP showing sexually dimorphic projections in the glomeruli from *fru* ORNs (DA1, VA1v, VL2a), projections in the antennal mechanosensory motor complex (AMMC) from *fru* JONs, sexually dimorphic projections in the prothoracic neuromere from *fru* LAN1 in the forelegs, and sexually dimorphic arbors in the abdominal neuromere (Abg). Tissues were stained with anti-mCD8 (green) and anti-nC82 (magenta). Scale Bar = 50 μm. **(B)** Expression profile of fru-GAL4, UAS>stop>mCD8::GFP, FLP^335^, tsh-GAL80. The addition of tsh-Gal80 transgene resulted in less expression in the ventral cord, particularly at the abdominal ganglion. **(F,G)** Magnified image of abdominal ganglion showing *fru* neurons. Immunofluorescence is reduced with the introduction of tsh-Gal80 **(G)**. Tissues were stained with anti-mCD8 (green) and anti-nC82 (magenta). Scale Bar = 50 μm. **(C–E)** Effect of silencing FLP^335^ restricted labeling of fru-Gal4 with and without tsh-GAL80. **(C)** Percentage of successful matings in 1 h (FLP^335^, fru>TNTin: *n* = 33; FLP^335^, fru>TNT: *n* = 48; FLP^335^, fru>TNTin, tsh-GAL80: *n* = 24; FLP^335^, fru>TNT, tsh-GAL80: *n* = 40). ^∗∗∗^*p* = 0.0001, ^∗∗∗^*p* = 0.0004 by Fisher’s exact test. **(D)** Copulation latency (central line indicates the median; FLP^335^, fru>TNTin: *n* = 27; FLP^335^, fru>TNT: *n* = 10; FLP^335^, fru>TNTin, tsh-GAL80: *n* = 22; FLP^335^, fru>TNT, tsh-GAL80: *n* = 28). ^∗∗∗^*p* = 0.0002, n.s. = not significant by Mann–Whitney test. **(E)** Copulation duration (central line indicates the median; FLP^335^, fru>TNTin: *n* = 27; FLP^335^, fru>TNT: *n* = 16; FLP^335^, fru>TNTin, tsh-GAL80: *n* = 22; FLP^335^, fru>TNT, tsh-GAL80: *n* = 28). ^∗∗∗^*p* < 0.0001, ^∗∗^*p* = 0.0041 by Mann–Whitney test.

We focused on one *FLP* line, *FLP^335^*, in the low fertility category that showed consistent restrictive labeling of several sexually dimorphic arborizations. Specifically, FLP^335^, fru>mCD8::GFP showed robust labeling of three classes of sensory projections that send sexually dimorphic projections into the CNS: (1) *fru* ORNs that project to the sexually dimorphic glomeruli (DA1, VA1v, and VL2a), which have been shown to be significantly larger in males (Stockinger et al., 2005); (2) *fru* GRNs (LAN1) that send sexually dimorphic midline crossing arbors into the contralateral prothoracic neuromere (Mellert et al., 2010; Yu et al., 2010); and (3) *fru* expression at the abdominal ganglion that consists of 13.4 ± 4.0 (*n* = 22) neurons and arborizations that may originate from the peripheral tissues (**Figures 2A,F**). Labeling of some neurons in the optic lobes (~67% of all dissected samples; *n* = 21) and a subset of Kenyon cells and their projections (~57% of all dissected samples; *n* = 21) was also observed, but they were less consistent (**Table 1**). In the ventral nerve cord, a few additional labeled neurons in the prothoracic ganglion were observed (**Figure 2A**). However, the labeling patterns of these neurons varied across individual preparations.

**Table 1:** Summary of all genetic combinations, the corresponding expression pattern, and copulatory behavior discussed in this paper.

Transgenes		Expression Characterization with GFP	Functional Characterization by Neuronal Silencing with TNT		
GAL4	tsh-GAL80		Percent Copulated in 1 h	Percent Change in Latency to Copulate Compared to Control (TNT vs. TNTin)	Percent Change in Copulation Duration Compared to Control (TNT vs. TNTin)
fru	-	Sporadic expression in the optic lobes (~67% of all dissected samples, *n* = 21), MB (~57% of all dissected samples, *n* = 21) and, consistent expression of the glomeruli, AMMC, GRNs, Abg (13.4 ± 4.0 neurons in the abdominal ganglion, and arborizations from peripheral tissues)	~30%^∗∗∗^	↑~300%^∗∗∗^	↓25%^∗∗∗^
fru	+	Sporadic expression in the MB, Glomeruli, AMMC, GRNs, Abg (8.9 ± 2.5, *n* = 14 neurons in the abdominal ganglion and arborizations from peripheral tissues)	~70%^∗∗∗^	NS	↓22%^∗∗^
Orco		80% of all ORNs (Wang et al., 2003)	92%	NS	NS
Ppk23		GRN specific (Thistle et al., 2012)	100%	NS	NS
TRH	+	sAbg-1 (4 ± 1, *n* = 6 neurons in the abdominal ganglion)	100%	NS	↓10%^∗∗^
TH	+	One pair of neurons in the anterior lateral protocerebral region of the brain, sAbg-1 (4 ± 2, *n* = 5 neurons in the abdominal ganglion)	100%	NS	↓9%^∗^

*^∗∗∗^p < 0.0005, ^∗∗^p < 0.005, ^∗^p < 0.05, n.s., not significant. MB, mushroom body; AMMC, antennal mechanosensory motor complex; GRN, gustatory receptor neurons.*

Consistent with the low fertility observed in our initial screen, FLP^335^, fru>TNT performed poorly in classical courtship assays as indicated by a significant reduction in the CI compared to controls (an inactive form of TNT, TNTin) (data not shown). Only 33% of males showed successful copulation within 1 h. Compared to controls, the latency to copulate was three times as long, and the copulation duration was significantly reduced by 25% (**Figures 2C–E** and **Table 1**). The low courtship intensity is likely due to the silencing of the sensory afferents.

To determine which targeted neurons are responsible for the observed courtship defects, we incorporated the *tsh-GAL80* transgene to suppress GAL4 activity in the ventral nerve cord. *tsh* is an essential gene that determines the cellular identity of the ventral nerve cord (Fasano et al., 1991; Roder et al., 1992). The *tsh-GAL80* was generated (Simpson, 2016) and had been used to silence *GAL4* activity in the ventral nerve cord by multiple groups (Clyne and Miesenbock, 2008; Zhang et al., 2013; Andrews et al., 2014; Harris et al., 2015). The resulting males (FLP^335^, fru>mCD8::GFP, tsh-GAL80) retained most of the original GFP expression pattern in the central brain region. However, in the ventral nerve cord and abdominal ganglion, limited reduction in GFP expression was observed, with only 8.9 ± 2.5 (*n* = 14) neurons showing expression (**Figures 2B,G**, and **Table 1**). The addition of tsh-GAL80 eliminated some of the variability in GFP expression in the ventral nerve cord, particularly those outside of the posterior tip of the abdominal ganglion. Silencing these neurons (FLP^335^, fru>TNT, tsh-GAL80) resulted in a partial rescue of the copulation success and complete rescue of the latency phenotype, but the copulation duration was still significantly reduced (**Figures 2C–E**). Therefore, the copulation duration phenotype may be due to the silencing of the remaining neurons at the posterior tip of the abdominal ganglion or the peripheral neurons that project to the area.

However, FLP^335^, fru>TNT, tsh-GAL80 males also showed robust targeting of the *fru* ORNs, JONs, and GRNs. In order to rule out their contribution to the copulation phenotype, we swapped the *fru*-*GAL4* line with other *GAL4* lines that are known to specifically label these neuropils. Silencing the ORNs (FLP^335^, orco>TNT) (Wang et al., 2003) or GRNs (FLP^335^, ppk23>TNT) (Thistle et al., 2012) in males had little impact on the copulatory phenotypes (Supplementary Figure 1 and **Table 1**). We did not account for the effect of the antennal mechanosensory motor complex arborization projecting from the JONs, which is the auditory organ in flies, as hearing is dispensable for pre-copulation behavior (Markow, 1987), and unlikely to play a role in post-copulation behavior.

### Copulation Duration Is Regulated by Sexually Dimorphic Serotonergic *fru* Neurons (sAbg-1) at the Posterior Tip of the Abdominal Ganglion

**FIGURE 3:**
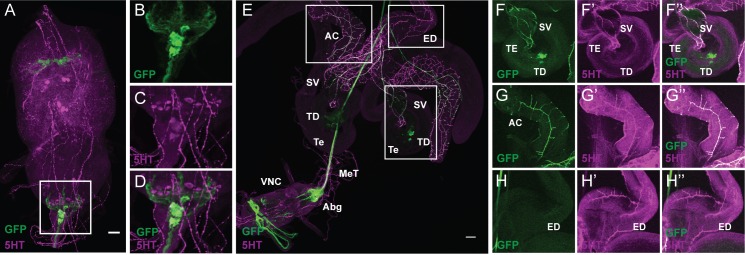
FLP^335^ restricted expression of fru-GAL4 with tsh-GAL80 targeted a subset of abdominal serotonergic fru neurons. **(A–D)** Double staining of male VNC with anti-GFP (green) and anti-5HT (magenta) of a Z-stack projection. **(E)** Double staining of the male reproductive organs with anti-GFP (green) and anti-5HT (magenta). **(F–H)** Co-localization of GFP and 5HT signal at the seminal vesicle **(F)**, the accessory glands **(G)**, and the absence of GFP signal at the ejaculation duct **(H)**. AC, accessory gland; ED, ejaculation duct; SV, seminal vesicle; TD, testicular duct; Te, testes; MeT, median trunk nerve; VNC, ventral nerve cord; Abg, abdominal ganglion. Scale Bar = 50 μm. The brightness of **E** had been adjusted in ImageJ so that the innervation at the peripheral tissues can be visualized [**B**: Mean Intensity in Red/Blue Channel (displayed as magenta) = 38.06 ± 41.28, Mean Intensity in Green Channel = 6.63 ± 15.75; **E**: Mean Intensity in Red/Blue Channel (displayed as magenta) = 20.53 ± 31.66, Mean Intensity in Green Channel = 11.62 ± 24.89].

We identified a subset of *fru* neurons in the abdominal ganglion that are likely to be involved in the regulation of copulation duration. We next proceeded to identify the signaling mechanism of these neurons by using antibodies to label various neurotransmitters. We found that the subset of *fru* neurons in the abdominal ganglion are immunoreactive to antibodies against serotonin (5HT) (**Figures 3A–D**). Early characterization of *fru* neurons in the abdominal ganglion had previously identified 8–10 male-specific neurons that are immunoreactive to 5HT (s-Abg) (Lee and Hall, 2001; Lee et al., 2001; Billeter and Goodwin, 2004). With cell bodies located at the posterior tip of the abdominal ganglion, s-Abg sends axons through the MeT to the junction of the reproductive tissues that include pairs of AC, SVs, and the anterior end of the ED (Lee and Hall, 2001; Lee et al., 2001; Billeter and Goodwin, 2004). Indeed, immunostaining of FLP^335^, fru>mCD8::GFP, tsh-GAL80 males with anti-5HT confirmed that most of the targeted *fru* abdominal neurons were serotonergic (~86%, *n* = 6). As previously reported, these neurons send axons via the MeT to innervate the SV (**Figures 3E,F**) and the AC (**Figures 3E,G**). However, we did not observe GFP expression in the ED (**Figures 3E,H**). Therefore, the *FLP^335^, fru-GAL4, tsh-GAL80* genetic combination targets a subset of s-Abg innervation at the male reproductive tissues. Hereafter, we will refer to this subset of serotonergic *fru* neurons as sAbg-1.

**FIGURE 4:**
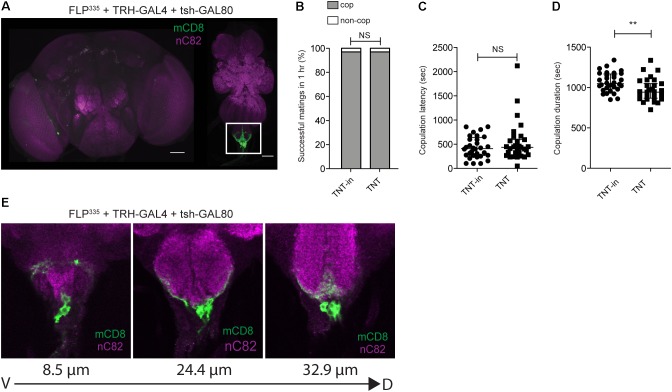
FLP^335^ and tsh-GAL80 restrictive labeling of the serotonergic GAL4 line. **(A)** Expression profile of FLP^335^ tsh-GAL80 TRH-GAL4 UAS>stop>mCD8::GFP showing exclusive targeting of neurons in the abdominal ganglion. **(E)** Magnified images of the abdominal ganglion at three different depths showing serotonergic cells. Tissues were stained with anti-mCD8 (green) and anti-nC82 (magenta). Scale Bar = 50 μm. **(B–D)** Effect of silencing FLP^335^ and tsh-GAL80 restrictive labeling of TRH-GAL4. **(B)** Percentage of successful matings in 1 h (FLP^335^, TRH>TNTin, tsh-GAL80: *n* = 33; FLP^335^, TRH>TNT, tsh-GAL80: *n* = 32). NS, not significant by Fisher’s exact test. **(C)** Copulation latency (central line indicates the median; FLP^335^, TRH>TNTin, tsh-GAL80: *n* = 32; FLP^335^, TRH>TNT, tsh-GAL80: *n* = 31). NS, not significant by Mann–Whitney test. **(D)** Copulation duration (central line indicates the median; FLP^335^, TRH>TNTin, tsh-GAL80: *n* = 32; FLP^335^, TRH>TNT, tsh-GAL80: *n* = 31). ^∗∗^*p* < 0.0019 by Mann–Whitney test.

As *FLP^335^*,*fru>TNT, tsh-GAL80* targets many neurons other than sAbg-1, to further confirm our hypothesis that sAbg-1 is responsible for the copulation duration phenotype, we replaced the *fru*-*GAL4* transgene with the specific *GAL4* transgene that targets the serotonergic system (*TRH*-*GAL4*). Colocalization of TRH>GFP and 5HT had been previously characterized (Alekseyenko et al., 2010). While most TRH+ cells in the abdominal ganglion do overlap with 5HT staining, we observed 7 ± 3 (*n* = 5) TRH- cells that stain with anti-5HT (Supplementary Figures 5E,F). The new genetic combination *FLP^335^, TRH > mCD8::GFP, tsh-GAL80* should recapitulate the sAbg-1 expression pattern in the abdominal ganglion but demonstrate distinct patterns elsewhere. Compared to *fru-GAL4*, gene expression targeted by *TRH-GAL4* in combination with *FLP^335^* was much more restrictive, with no expression in the brain (**Figure 4A**). In the ventral cord, GFP expression was only observed in 4 ± 1 (*n* = 6) neurons in the abdominal ganglion (**Figures 4A,E**). We silenced these neurons by replacing GFP with TNT, and once again, performed copulation assays. Compared to the inactive TNT controls, FLP^335^, TRH>TNT, tsh-GAL80 males had a normal copulation success rate and latency, but copulation duration was still significantly reduced by ~10% (**Figures 4B–D** and **Table 1**). Collectively, these results show that the copulation duration phenotype is regulated by sAbg-1, which is a subset of the previously characterized serotonergic *fru* cluster in the abdominal ganglion.

### The Copulation Time Regulating sAbg-1 Neurons Are Also Dopaminergic

**FIGURE 5:**
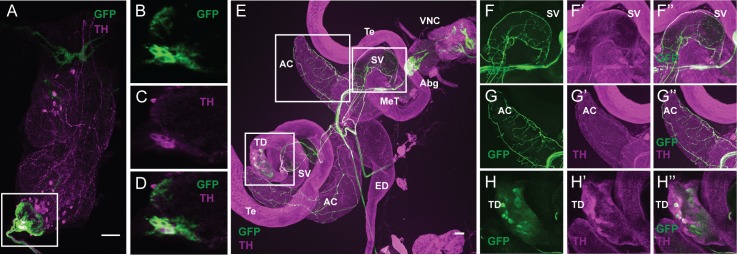
The serotonergic sAbg-1 neurons are also dopaminergic. **(A–D)** FLP^335^ restricted expression of fru-GAL4 with tsh-GAL80. Double staining of the male VNC with anti-GFP (green) and anti-TH (magenta) of a Z-stack projection. **(E)** Double staining of the male reproductive organs with anti-GFP (green) and anti-TH (magenta). **(F–H)** Co-localization of GFP and TH signals at the seminal vesicle **(F)**, the accessory glands **(G)**, and some cell-like structure near the testicular duct at the junction between the testes and the seminal vesicle **(H)**. AC, accessory gland; ED, ejaculation duct; SV, seminal vesicle; TD, testicular duct; Te, testes; MeT, median trunk nerve; VNC, ventral nerve cord; Abg, abdominal ganglion. Scale Bar = 50 μm.

In our neurotransmitter antibody screening experiment with FLP^335^, fru>mCD8::GFP, tsh-GAL80 male tissues, we observed that most of the cell bodies of sAbg-1 (~79%, *n* = 8) are also immunoreactive to the dopamine (DA)-synthesizing enzyme, TH (**Figures 5A–D**). For comparison, without the FLP^335^ and tsh-GAL80 transgenes, TRH>GFP labels 34.3 ± 10 (*n* = 3) TRH+ cells in the abdominal ganglion, most (99%) of which showed colocalization with anti-TH (Supplementary Figures 5A,B). Although much weaker than 5HT staining, immunoreactivity to the TH antibody was observed at the axons of the MeT nerve (**Figures 5E,F**) that extended to innervate the AC (**Figures 5E,G**) and the SV (**Figures 5E,F**). Moreover, some cell-like TH+ expression was observed at the junction between the testes and SV (**Figure 5E**, near label TD, **Figure 5H**), but these cells did not co-stain with anti-5HT (**Figure 3F**). However, they did show GFP expression in the FLP^335^ restricted serotonergic reproductive tissues (FLP^335^, TRH>mCD8::GFP, tsh-GAL80) (Supplementary Figures 2A–D). Therefore, the negative staining of 5HT could reflect the low level of 5HT in these cells since *TRH-GAL4* do target these cells. Finally as expected, all the sAbg-1 cells targeted by the *FLP^335^* and *TRH-GAL4* are also TH+ (Supplementary Figures 3A–M).

**FIGURE 6:**
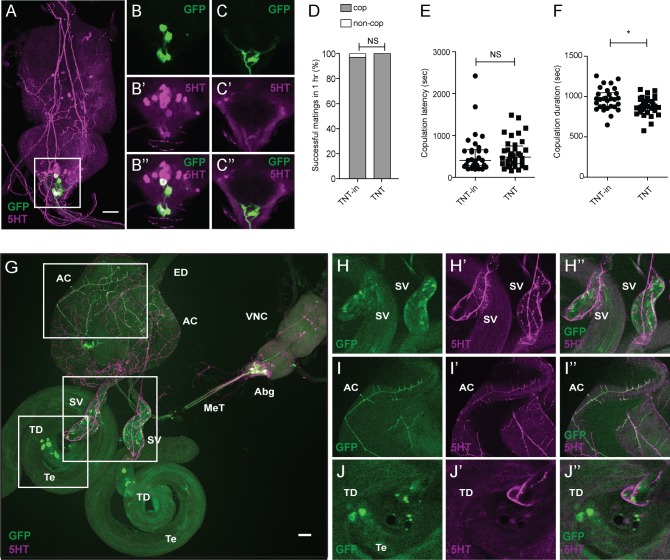
FLP^335^ and tsh-GAL80 restrictive labeling of the dopaminergic GAL4 line. **(A)** FLP^335^ tsh-GAL80 TH-GAL4 UAS>stop>mCD8::GFP showing exclusive targeting of neurons that are double positive for GFP and 5HT at the abdominal ganglion. **(B,C)** Two different depths are shown to illustrate the co-localized signals. Tissues were stained with anti-mCD8 (green) and anti-5HT (magenta). Scale Bar = 50 μm. **(D–F)** Effect of silencing FLP^335^ and tsh-GAL80 restrictive labeling of TH-GAL4. **(D)** Percentage of successful matings in 1 h (FLP^335^, TH>TNTin, tsh-GAL80: *n* = 31; FLP^335^, TH>TNT, tsh-GAL80: *n* = 36). NS, not significant by Fisher’s exact test. **(E)** Copulation latency (central line indicates the median; FLP^335^, TH>TNTin, tsh-GAL80: *n* = 30; FLP^335^, TH>TNT, tsh-GAL80: *n* = 36). NS, not significant by Mann–Whitney test. **(F)** Copulation duration (central line indicates the median; FLP^335^, TH>TNTin, tsh-GAL80: *n* = 30; FLP^335^, TH>TNT, tsh-GAL80: *n* = 36). ^∗^*p* < 0.0172 by Mann–Whitney test. **(G)** Double staining experiment of the FLP^335^ tsh-GAL80 restricted GFP expression of TH-Gal4 male reproductive tissues with anti-GFP (green) and anti-5HT (magenta). **(H–J)** Co-localization of GFP and 5HT signals at the seminal vesicle **(H)**, the accessory glands **(I)**, and the cell-like structure near the testicular duct at the junction between the testes and the seminal vesicle **(J)**. AC, accessory gland; ED, ejaculation duct; SV, seminal vesicle; TD, testicular duct; Te, testes; MeT, median trunk nerve; VNC, ventral nerve cord; Abg, abdominal ganglion.

To confirm that sAbg-1 is also dopaminergic, we investigated whether the *FLP^335^* restricted dopaminergic system would recapitulate sAbg-1 expression and lead to the same copulation phenotype when silenced by *TNT*. Interestingly, *FLP^335^, TH>mCD8::GFP, tsh-GAL80* targeted only 4 ± 2 (*n* = 5) neurons in the ventral cord (**Figure 6A**). For comparison, without the FLP^335^ and tsh-GAL80 transgenes, TH>GFP in the abdominal ganglion showed 37.3 ± 10 (*n* = 4) TH+ cells, 6 ± 2 (~16%) showed colocalization with anti-5HT (Supplementary Figures 5C,D). Consistent with previous observations, these neurons showed GFP and 5HT co-localization, suggesting that they are the same population as the sAbg-1 neurons (**Figures 6B,C**). The expression level of 5HT in these cells is variable since cells located more posterior are weaker and cells that are more anterior show very intense staining (**Figures 6A–C**). As expected, co-localization was also observed in the sAbg-1 axons at the MeT, and TH+ neuronal innervation was observed at the same male reproductive tissues as described above (**Figures 6G–J**). Almost no expression was observed elsewhere in the nervous system (Supplementary Figure 4). When sAbg-1 restricted by *TH-GAL4* and *FLP^335^* was silenced by TNT, *FLP^335^, TH>TNT, tsh-GAL80* males had a normal copulation success rate and latency, but copulation duration was still significantly reduced by ~9% (**Figures 6D–F** and **Table 1**). Taken together, these results show that the copulation duration phenotype we observed is regulated by sAbg-1, which utilizes both 5HT and DA as co-transmitters.

## Discussion

Classic *fru* mutants have different degrees of chromosomal lesions, resulting in different severity of courtship defects that correlate with the number of *fru* neurons expressing Fru^M^ within the *fru* circuit (Lee and Hall, 2001). As the expression of Fru^M^ is required for the normal development of *fru* neurons, different genetic combination of *fru* mutant heterozygotes would have defects in different regions of the *fru* circuit. Copulation duration of these classic *fru* mutant heterozygotes is on average longer, and has greater variability than that of wild-type flies (Lee et al., 2001). This suggests that the regulation of copulation time may involve a time-setting mechanism, with some neurons controlling the lengthening and others controlling the shortening of the duration. Together, these *fru* neurons function to ensure that copulation duration is tightly controlled. Here, we identified a small subset of ~4 *fru* neurons (sAbg-1) at the posterior tip of the abdominal ganglion that regulate copulation duration. When sAbg-1 is silenced by TNT, copulation duration is shortened. sAbg-1 is a subset (~50%) of the previously characterized serotonergic *fru* neurons at the abdominal ganglion, with similar innervation at the SV, AC, and near the testicular duct, but not the ED (Lee and Hall, 2001; Lee et al., 2001; Billeter and Goodwin, 2004). We discovered that sAbg-1 neurons are both serotonergic and dopaminergic, and confirmed this observation in four sets of experiments, making use of intersectional genetics, behavioral assays, and immunostaining.

The discovery that sAbg-1 neurons are both serotonergic and dopaminergic is surprising. Although evidence of neurotransmitter co-release has been previously described, most reported cases involve fast excitatory and inhibitory neurotransmitters or fast neurotransmitters coupled with slow neuromodulators (Hnasko and Edwards, 2012; Saunders et al., 2015). Co-release can be due to the ability of the VMAT to take up neurotransmitters released by another neuron. For example, striatal DA terminals in mammalian brain slices have been found to co-release both DA and 5HT (Zhou et al., 2005). The availability of 5HT released by a dopaminergic neuron is due to the non-specific uptake of excess 5HT, which accumulates at the DA terminals, by DA transporters that have low affinity for 5HT (Zhou et al., 2005). In the current case, the behavioral and anatomical evidence indicates that sAbg-1 neurons have the biosynthetic enzymes to synthesize both DA and 5HT. In *Drosophila*, VMAT is required for the storage and release of neurotransmitters in all aminergic neurons. Interestingly, when VMAT is expressed in the serotonergic system in a VMAT-null background, an increased level of DA is observed (Chen et al., 2013), suggesting an interaction or redundancy of the two aminergic systems in *Drosophila*. The physiological significance of the co-release of 5HT and DA from these neurons to modulate copulation duration is unclear. It is possible that the release of 5HT or DA is initiated by different signals. The relative activation of their cognate postsynaptic receptors may be one mechanism which regulates copulation duration.

Serotonergic *fru* neurons, to which sAbg-1 belongs, have been reported to express the receptor for the neuropeptide corazonin (CrzR). These neurons (*fru*/5HT/CrzR) receive inputs from a cholinergic subset of abdominal *fru* neurons that express corazonin. Upon activation, *fru*/5HT/CrzR neurons stimulate sperm transfer (Tayler et al., 2012). The *fru*/5HT/CrzR neurons are proposed to set the lower time limit of copulation duration, as their activation stimulates sperm ejaculation, and shortens copulation to only ~7 min (Tayler et al., 2012). Unlike the silencing of sAbg-1, which led to a shortened copulation duration, silencing of *fru*/5HT/CrzR by the expression of the inward rectifying potassium channel K_ir_, using either CrzR-GAL4 or Tph2-GAL4 (generated the same way as our TRH-GAL4), resulted in a normal copulation duration (Tayler et al., 2012). The difference is likely because of the additional silencing of the ED or other tissues that may be innervated by the other *fru* serotonergic neurons not part of sAbg-1.

Intriguingly we also observed that the *fru* neurons targeted by FLP^335^ tsh-GAL80 fru-GAL4 UAS>stop>TNT, which are almost all 5HT and TH positive, are necessary for normal fertility in addition to copulation duration (data not shown). It is not surprising given that these neurons innervate most of the male reproductive organs. However, we have also observed in other FLP lines where a reduction in copulation duration does not affect fertility. Thus, copulation duration regulated by *fru* neurons have different physiological purposes.

Copulation duration is multifactorial, and other mutants have been found to affect this parameter. Feminizing *engrailed* [encoding Engrailed (En)]-expressing *fru* neurons in the CNS result in a wide variation of copulation duration (Latham et al., 2013). These *fru*/En neurons within the abdominal ganglion use γ-aminobutyric acid, and therefore, are distinct from the sAbg-1 cluster described here (Latham et al., 2013). Null mutants of the clock genes *per* and *tim* have been reported to lengthen copulation duration, independent of the core clock mechanism (Beaver and Giebultowicz, 2004). We cannot rule out the involvement of *per* and *tim* playing a non-circadian regulatory role in the copulation phenotype that we observed.

The neural regulation of copulatory behaviors involves multiple neural circuits ranging from sensory inputs to motor controls. The systematic functional characterization of the *fru* circuit allows us to understand the neural mechanisms underlying copulatory behaviors. Our finding contributes to a better understanding of a complex neural circuit.

## Materials and Methods

### Fly Stocks

The following strains were used in this study: *fru-GAL4* (Stockinger et al., 2005), *UAS>stop>TNTin*, *UAS>stop>TNT* (Stockinger et al., 2005), *UAS>stop>mCD8::GFP* (Yu et al., 2010), *FLP* from Liqun Luo (Department of Biology, Stanford University, CA, United States), *tsh-GAL80* from Julie Simpson, *TH-GAL4* from Serge Birman (Development Biology Institute of Marseille, Marseille, France), *TRH-GAL4* (Alekseyenko et al., 2010), *orco-GAL4, ppk23-GAL4*, *elav^c155^-GAL4*, and the Canton-S strain from the Bloomington Stock Center, Bloomington, IN, United States.

### Generation of Enhancer-Trap FLP Lines

Detailed procedures for the generation of the enhancer-trap FLP library has been described in a previous publication (Alekseyenko et al., 2013). A total of 356 enhancer-trap FLP lines (*FLP*) were generated by randomly inserting a FLP recombinase in chromosome II and III. Individual *FLP* lines were subsequently crossed to *elav^c155^-GAL4; UAS>stop>mCD8::GFP* to check for GFP expression. Approximately 200 lines that showed consistent expression in the nervous system in 1–3-day-old adult males were selected.

### Immunohistochemistry

Immunohistochemistry was performed on samples from 3 to 7-day-old adult flies. Dissection and immunohistochemistry of the adult nervous system were performed as described previously (Mundiyanapurath et al., 2009), with some modifications in the primary and secondary antibodies used. Dissection of male reproductive organs was performed on Sylgard plates (Living Systems Instrumentation, St. Albans City, VT, United States) covered with 0.1 M phosphate-buffered saline. Dissected samples were fixed, and immunostaining was performed as described. The following primary and secondary antibodies were used in this study: rat polyclonal anti-mCD8 (1:100; Caltag, Burlingame, CA, United States), rabbit anti-GFP (1:600; Thermo Fisher Scientific, Waltham, MA, United States), mouse anti-GFP (1:500, Thermo Fisher Scientific), mouse anti-nc82 (1:10; Developmental Studies Hybridoma Bank, Iowa City, IA, United States) (Hofbauer et al., 2009), rabbit anti-Fru^M^ (1:2000) (kindly supplied by Dickson laboratory), rabbit anti-TH (1:500; Novus Biologicals, Littleton, CO, United States; although the immunogen was derived from rat, its fly specific immunoreactivity has been reported in at least 4 publications (Venderova et al., 2009; Alekseyenko et al., 2010, 2013; Kuo et al., 2015), rabbit anit-5HT (1:500; Sigma-Aldrich, St. Louis, MO, United States), anti-rat IgG conjugated with Alexa Fluor 488 (1:200; Thermo Fisher Scientific), goat anti-mouse IgG conjugated with Alexa Fluor 647 (1:200; Thermo Fisher Scientific), and anti-rabbit IgG conjugated with Alexa Fluor 594 (1:200; Thermo Fisher Scientific).

### Imaging and Expression Analysis

Images of the *FLP* expression pattern were acquired by Eclipse 90i (Nikon, Minato, Tokyo, Japan) fluorescence microscope using a 20× water-immersion objective. Structured illumination microscopy (Optigrid) was used to obtain optical sections (1.8 μM) of the whole central brain region. ImageJ software (National Institutes of Health, Bethesda, MD, United States) was used to generate the Z-stack projection of the *FLP* expression pattern.

### Behavioral Assays

#### Husbandry

Flies were raised on standard cornmeal medium, and kept in a 12 h:12 h day:night cycle at 25°C in ambient relative humidity. Each newly enclosed adult was collected and aged for 3–7 days in an isolation vial (16 × 100-mm) supplied with ~2 ml of fly food. Virgin, wild-type Canton-S females were aged in groups of 20–40 for 3–7 days. All behavioral experiments were performed at 25°C with ~50% humidity, during the first 3 h after lights on.

#### Fertility Screen

The fertility screen was performed by adding a virgin Canton-S female to the isolation vial where the test male had been isolated. The pairs were allowed to interact for ~30 min, after which time the males were removed. After 4 (or more) days, any progeny from the pairing was recorded. The number of pairings that successfully produced progeny over the total number of pairings for each genotype is defined as the FI (*n* > 10).

#### Courtship Behavior

Courtship assays were performed using a chamber wheel with four compartments made of acrylic glass. Each compartment has a diameter of 3 cm and a height of 1 mm. A test male and a virgin female was each aspirated to each compartment, and video-recorded. The CI is the percentage of time the male spent courting the female in the 10 min after the female is added to the chamber (Villella and Hall, 2008). The courtship vigor index is similar to CI except the 10-min observation period begins after the first courtship event, thus delineating courtship latency from courtship intensity (Krstic et al., 2009). Copulation duration is defined as the duration of the complete copulation event. Latency to court is the time taken when the first courtship event is observed. Latency to copulate is the time taken when copulation is successful.

#### Copulation Assay

Copulation assays were performed in 12-well plates (Falcon, Corning Inc., Corning, NY, United States) with ~2 ml standard fly food to maintain humidity in each well. An experimental male was paired with a virgin Canton-S female, and the courtship behavior was video-recorded for at least 1 h. Any pair that did not copulate during a 1-h period was considered unsuccessful in copulation, and was not included in the calculation of copulation latency or duration. Copulation latency was determined to be the time from the beginning of pairing to the time at which successful copulation was observed. Copulation duration was measured from the start of the first successful mounting to the dismounting of the male from the female.

#### Statistical Analysis

All statistical analyses were performed using GraphPad Prism software (version 5.0b) For mating success experiments, the percentage of males of each genotype that succeeded in mating with a wild type virgin female within a 1-h window was compared using Fisher’s exact test (**Figures 2C**, **4B**, **6D** and Supplementary Figure 1A) (^∗^*p* < 0.01, ^∗∗^*p* < 0.005, ^∗∗∗^*p* < 0.0001). For all copulation latency and copulation duration tests described, values obtained for experimental males where compared with those of control males using a Mann–Whitney test (**Figures 2D,E**, **4C,D**, **6E,F** and Supplementary Figures 1B,C) (^∗^*p* < 0.01, ^∗∗^*p* < 0.005, ^∗∗∗^*p* < 0.0001).

## Author Contributions

AL and YC conceived, designed, and performed the experiments. AL wrote the manuscript with support from MF. SJ performed the experiments. All authors provided critical feedback and helped design the research, analysis, and manuscript.

## Conflict of Interest Statement

The authors declare that the research was conducted in the absence of any commercial or financial relationships that could be construed as a potential conflict of interest.

## Abbreviations

5HT: serotonin
AC: accessory gland
CI: courtship index
CrzR: corazonin receptor
DA: dopamine
ED: ejaculation duct
En: engrailed
FI: fertility index
FLP: enhancer trap-FLP recombinase
fru: fruitless
GFP: green fluorescent protein
GRN: gustatory receptor neuron
MeT: median trunk nerve
ORN: olfactory receptor neuron
per: period
SV: seminal vesicle
TH: tyrosine hydroxylase
tim: timeless
TNT: tetanus toxin light chain
VMAT: vesicular monoamine transporter
