# Supplementary material for: Characterization of the Sexually Dimorphic fruitless Neurons That Regulate Copulation Duration
Source: Front Physiol. 2018 Jun 25;9:780. doi: 10.3389/fphys.2018.00780 (PMC6026680; doi:10.3389/fphys.2018.00780)
Supplement: Supplementary file 1 [file Presentation_1.pdf]

*Supplementary Material*

# Characterization of the Sexually Dimorphic *fruitless* Neurons that Regulate Copulation Duration

**Shreyas Jois[1#], Yick-Bun Chan[2#], Maria Paz Fernandez[2], Adelaine Kwun-Wai Leung[1*]**

*** Correspondence:** Adelaine Kwun-Wai Leung: adelaine.leung@usask.ca

# Co-first author

## 1.1 Supplementary Figures

frontiers

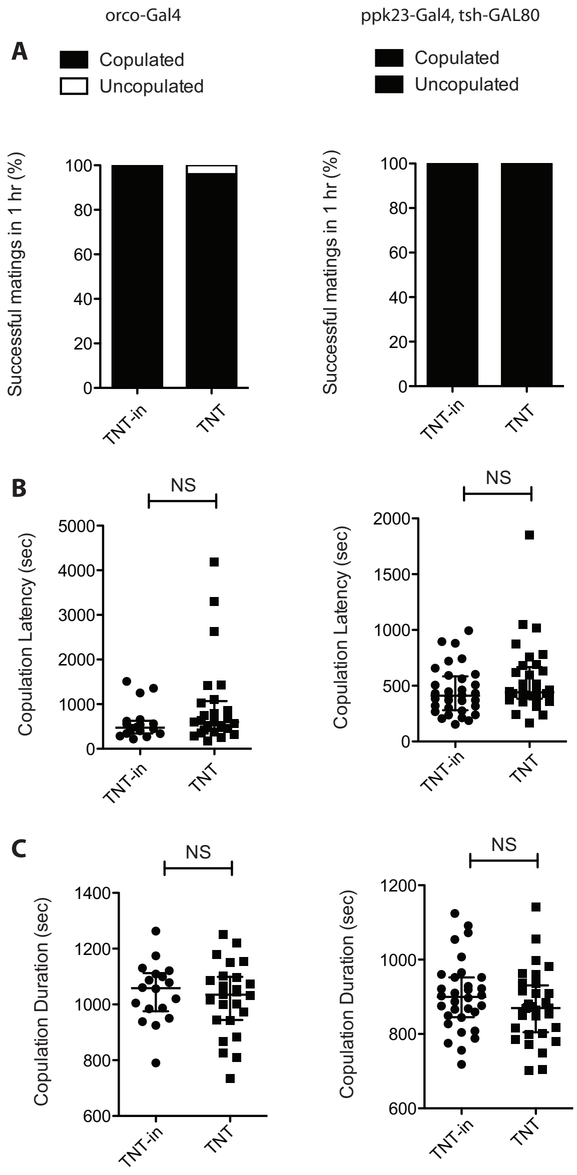


**Supplementary Figure 1.** Silencing the olfactory receptor neurons and gustatory receptor neurons targeted by FLP$^{335}$ restricted labeling of orco-GAL4 and ppk23-GAL4 had no influence on copulatory behaviours. (A) Percentage of successful matings in 1 hr (Control=FLP$^{335}$, orco>TNTin : n=32 and FLP$^{335}$, ppk>TNTin, tsh-GAL80: n=18; FLP$^{335}$, orco>TNT: n=26; FLP$^{335}$, ppk23>TNT, tsh-GAL80: n=32). (B) Copulation duration (central line indicates the median; Control=FLP$^{335}$, orco>TNTin: n=32 and FLP$^{335}$, ppk>TNTin, tsh-GAL80: n=18; FLP$^{335}$, orco>TNT: n=24; FLP$^{335}$, ppk23>TNT, tsh-GAL80: n=32). (C) Copulation latency (central line indicates the median; Control=FLP$^{335}$, orco>TNTin: n=32 and FLP$^{335}$, ppk>TNTin, tsh-GAL80: n=18; FLP$^{335}$, orco>TNT: n=25; FLP$^{335}$, ppk23>TNT, tsh-GAL80: n=32). NS= not significant by Fisher's exact test (A) and Mann-Whitney test (B and C).

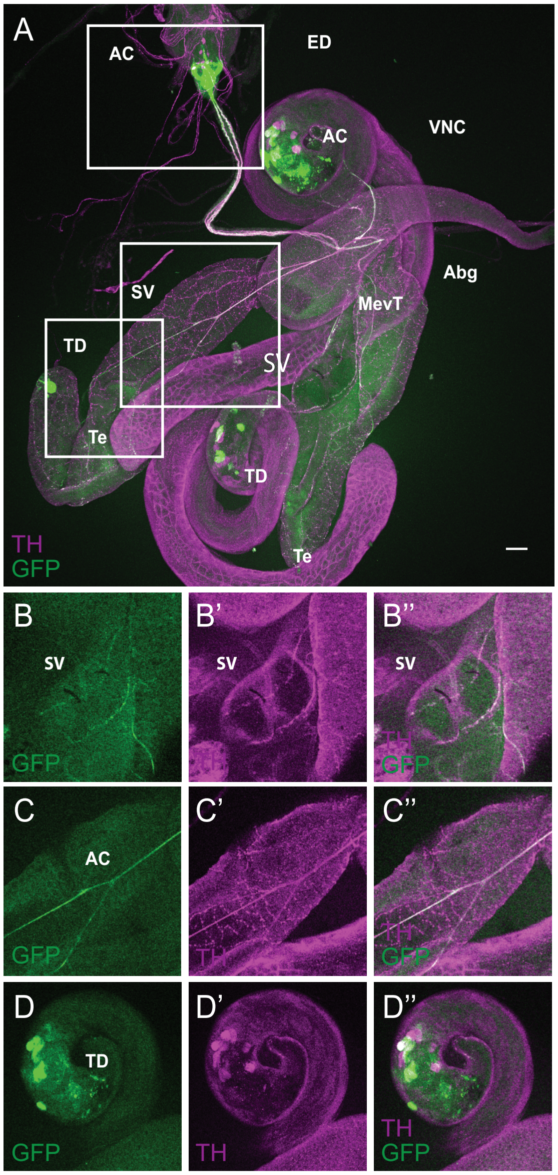


**Supplementary Figure 2. The serotonergic sAbg-1 neurons are also dopaminergic.** (A-D) $FLP^{335}$ restricted expression of TRH-GAL4 with tsh-GAL80. Double staining of the male reproductive organs with anti-GFP (green) and anti-TH (magenta). Co-localization of GFP and TH signals at the seminal vesicle (B), the accessory glands (C), and some cell-like structure near the testicular duct at the junction between the testes and the seminal vesicle (D). AC=accessory gland; ED=ejaculation duct; SV=seminal vesicle; TD=testicular duct; Te=testes; MeT=median trunk nerve; VNC=ventral nerve cord; Abg=abdominal ganglion. Scale Bar = 50 µm.

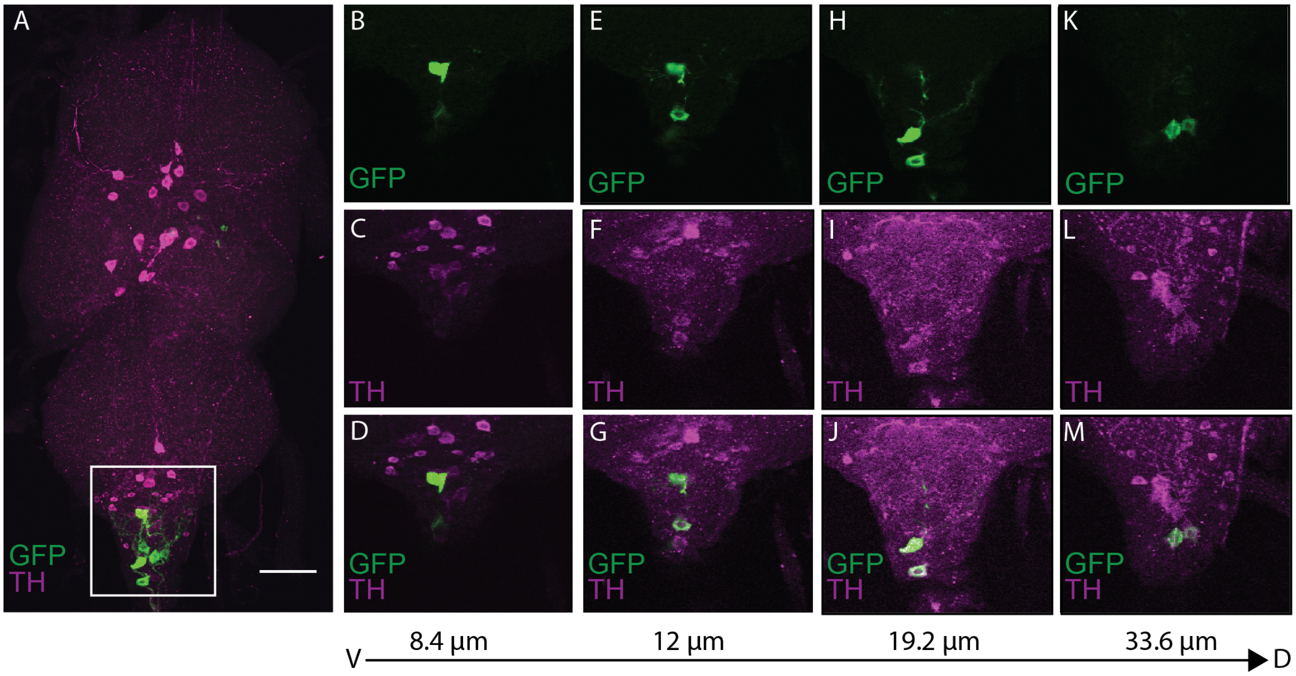


**Supplementary Figure 3. The sAbg-1 neurons in the abdominal ganglion co-express serotonin and dopamine.** (A) FLP$^{335}$ tsh-GAL80 TRH-GAL4 UAS>stop>mCD8::GFP showing exclusive targeting of neurons that are double positive for GFP and TH at the abdominal ganglion. (B-M) Four different depths are shown to illustrate the co-localized signals. Tissues were stained with anti-mCD8 (green) and anti-TH (magenta). V=Ventral; D=Dorsal. Scale Bar = 50 µm.

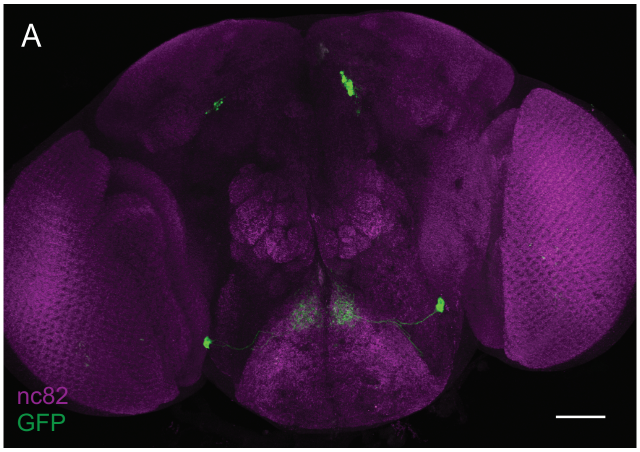


**Supplementary Figure 4.** (A) Male brain tissues of FLP$^{335}$ restricted expression of TH-GAL4 with tsh-GAL80 stained with anti-mCD8 (green) and anti-nC82 (magenta). Consistent GFP expression was observed only in a pair of neurons in the anterior lateral protocerebral region of the brain that project to the subesophageal ganglion. Scale Bar = 50 µm.

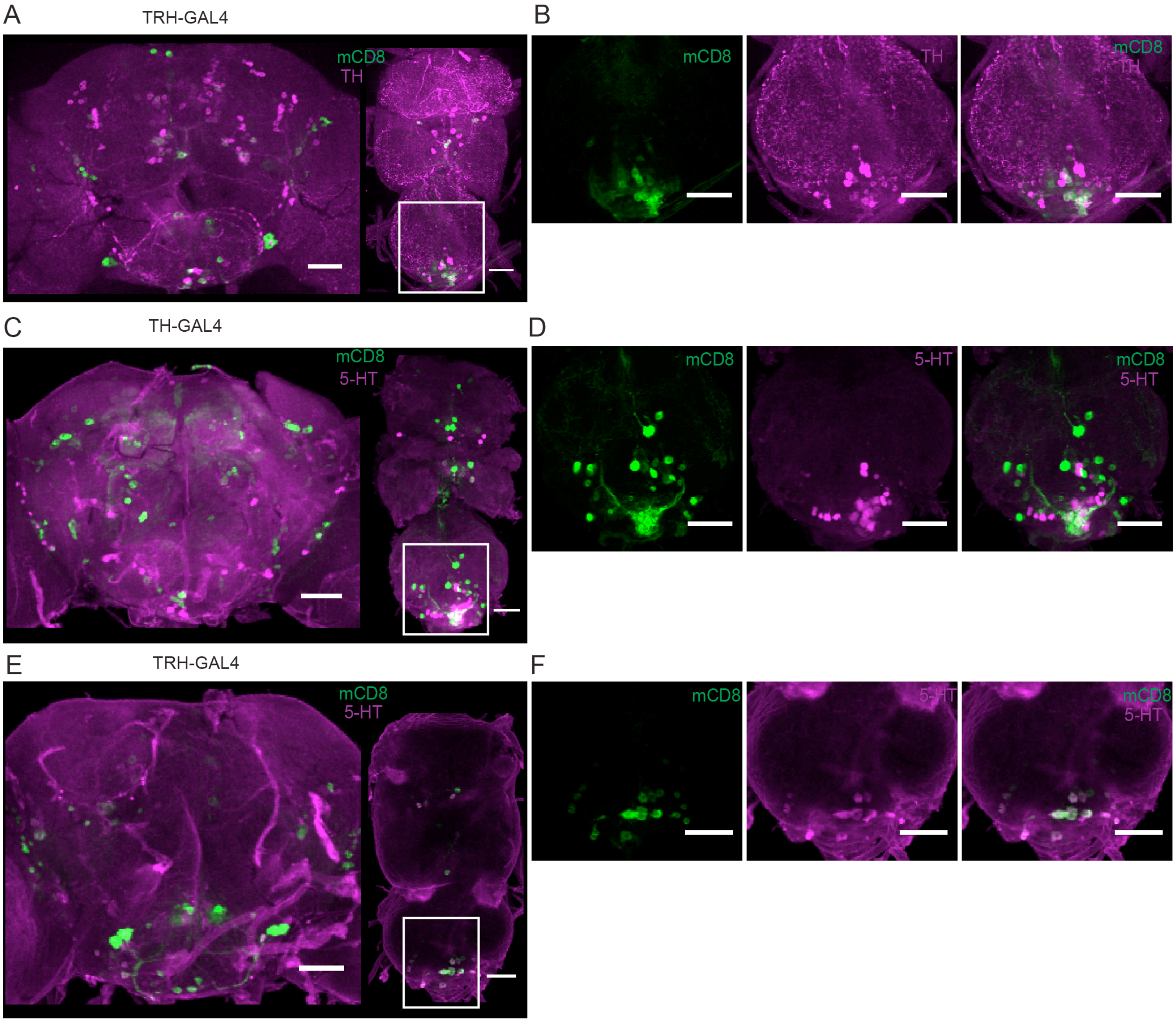


**Supplementary Figure 5.** (A) TRH-Gal4 expression pattern (green) and colocalization with anti-TH antibody (magenta). (B) Magnified view of colocalized serotonergic and dopaminergic cells in the abdominal ganglion. (C) TH-Gal4 expression pattern (green) and colocalization with anti-5HT antibody (magenta). (D) Magnified view of colocalized serotonergic and dopaminergic cells in the abdominal ganglion. (E) TRH-Gal4 expression pattern (green) and colocalization with anti-5HT antibody (magenta). (F) Magnified view of colocalization of TRH-Gal4 positive and anti-5HT positive serotonergic cells in the abdominal ganglion. Scale Bar = 50 µm.
